# Near-infrared autofluorescence versus visual identification during parathyroidectomy: a systematic review and meta-analysis of surgical outcomes

**DOI:** 10.3389/fendo.2026.1875193

**Published:** 2026-10-01

**Authors:** Jack Joseph Collins, Luis Bouz Mkabaah, Eoin Patrick Kerin, James C. Lennon, Ailbhe Kenny, Sami Medani Abd Elwahab, Aoife J. Lowery, Michael J. Kerin

**Affiliations:** 1 Lambe Institute of Translational Research, University of Galway, Galway, Ireland; 2 Department of Breast and Endocrine Surgery, Galway University Hospital, Galway, Ireland

**Keywords:** near infrared autofluorescence, NIRAF, parathyroid, parathyroidectomy, primary hyperparathyroidism

## Abstract

**Introduction:**

Near-infrared autofluorescence (NIRAF) is an emerging adjunctive tool that facilitates intraoperative identification of parathyroid glands. Although it has shown promise in the setting of thyroidectomy, its role in parathyroidectomy for primary hyperparathyroidism (PHPT) remains unclear.

**Methods:**

A systematic review was conducted according to PRISMA guidelines. Four databases were searched to identify studies comparing NIRAF with conventional visual identification of parathyroid glands during parathyroidectomy. The primary outcome was cure rates and secondary outcomes included operative time and the number of parathyroid glands identified. Methodological quality was assessed using Cochrane RoB2 and the Newcastle-Ottawa tools. Meta-analysis was performed using a random effects model.

**Results:**

Three studies comprising of 522 patients were included: randomized controlled trials and one observational cohort study. Meta-analysis showed a non-significant improvement in cure rates with NIRAF (RR 1.06, 95% CI: 1.02-1.10, p = 0.21). Mean operative time was reduced by 2.1 minutes with NIRAF (95% CI -4.76-9.0, p = 0.55) and the mean number of parathyroid glands identified increased by 0.12 per patient (95% CI -0.21-0.46, p = 0.46). Neither of the secondary outcomes reached statistical significance. Heterogeneity was moderate to high across the outcomes.

**Conclusion:**

Although NIRAF has proved promising in the setting of thyroidectomy, in the setting of parathyroidectomy for PHPT it did not offer statistically significant improvements in surgical outcomes. While the technology appears safe and promising, its impact on cure rates remains uncertain. Larger, high quality randomized controlled trials (RCTs) are warranted to define the role of NIRAF in parathyroid surgery more clearly.

## Highlights

- This is a focused systematic review and meta-analysis focused solely on surgical outcomes of NIRAF in the setting of parathyroidectomy.
- Cure rates were slightly higher with NIRAF but did not reach statistical significance.
- There were slightly more parathyroid glands identified with NIRAF but this was not statistically significant.
- NIRAF does not appear to offer a significant reduction in operative time during parathyroidectomy.

## Introduction

Primary hyperparathyroidism (PHPT) is a prevalent endocrine disorder characterized by overproduction of parathyroid hormone (PTH), leading to hypercalcaemia and a range of systemic sequelae including renal disease and osteoporosis (1). The reported prevalence of PHPT lies between 0.2 % and 1.3 % globally, and is being increasingly diagnosed due to improved biochemical analysis (2). Surgical excision of the overactive parathyroid gland(s) (PGs) remains the only definitive cure. However, parathyroidectomy is technically challenging, and morbidity is nontrivial.

The success of parathyroid surgery is dependent on accurate intraoperative identification of the pathological PGs. This task can be difficult due to their small size, variable anatomy, and occasional embedding within adipose or fibrous tissue (3). Despite the supportive use of pre-operative localization with ultrasound, sestamibi scintigraphy and computed tomography imaging modalities, traditional reliance on visual inspection introduces subjectivity and is dependent on surgeon experience (4). Other intra-operative identification methods described include methylene blue injection, indocyanine green injection and Technetium 99m sestamibi (MIBI) with isotopic detection (5–7). Adjunctive methods such as intraoperative PTH (ioPTH) assays or frozen section histology offer confirmatory data, but they can be costly and time consuming (8). Inadequate identification or misidentification may lead to persistent PHPT if diseased tissue is missed or hypoparathyroidism if healthy glands are inadvertently removed (1).

More recently, near-infrared autofluorescence (NIRAF) has emerged as a promising adjunct for intraoperative parathyroid gland identification. NIRAF utilizes the intrinsic fluorescent properties of parathyroid tissue. Autofluorescence was first described by Sir David Brewster in the 19^th^ century. He discovered that parathyroid tissues contained “fluorophores” that cause the tissue to fluoresce (9). When excited by near-infrared light (commonly ~ 758–785 nm), parathyroid glands emit light in the 700–900 nm range. This enables real-time, label-free localization without tissue removal (10). In thyroidectomy settings, NIRAF has demonstrated a 95% sensitivity for parathyroid gland identification, and may reduce the risk of accidental injury or incomplete resections (11). NIRAF also holds particular promise in standardizing outcomes across surgeons of differing experience. By reducing the reliance on subjective visual identification NIRAF may decrease variability across operators (12). Parathyroid adenomas may exhibit a heterogenous NIRAF pattern with a bright cap however the interpretation of the pattern can be subjective and there are no large multicenter RCTs assessing their fluorescence pattern (13).

Despite these encouraging advances, the role of NIRAF specifically in parathyroidectomy remains incompletely defined. To date, there is no dedicated systematic review that focuses exclusively on NIRAF in parathyroid surgery. Given the technical demands, potential morbidity, and the variability in surgical environments, a rigorous synthesis is warranted.

The primary aim is to systematically review the existing evidence on NIRAF as an intraoperative tool in parathyroidectomy for PHPT, comparing its performance to conventional visual identification primarily in the context of cure rates and additionally in the context of operative times and parathyroid gland identification.

## Materials and methods

This systematic review was conducted according to the Preferred Reporting Items for Systematic Reviews and Meta-Analysis (PRISMA) guidelines (14). Ethics approval or informed consent were not required for this systematic review of previously published studies.

### Population, intervention, comparison and outcome

The PICO framework (15) was used to defined the research question which we sought to address in this study.

Population: Adults undergoing parathyroidectomy for primary hyperparathyroidism (PHPT).

Intervention: Intraoperative use of near-infrared autofluorescence (NIRAF) for parathyroid gland identification.

Comparison: Conventional visual identification alone during parathyroidectomy.

Outcomes: Primary outcomes included: (1) Cure rate (biochemical resolution of PHPT/absence of persistent disease). Secondary Outcomes included: (1) Operative time; and (2) number of parathyroid glands identified intraoperatively.

### Eligibility criteria

Inclusion criteria were as follows: (1) randomized controlled trials (RCTs) and cohort studies comparing the use of NIRAF with visual inspection during parathyroid surgery for PHPT; and (2) studies had to provide full-text manuscripts to warrant inclusion (3) studies had to assess the primary outcome. Exclusion criteria included: (1) case reports; (2) studies with insufficient outcome data; (3) studies not published in the English language; and (4) animal or preclinical studies.

### Search strategy

A formal systematic search of PubMed, Scopus, Embase, and Cochrane databases was performed by two investigators independently to identify relevant publications (J.J.C and L.B.M), the latest of which occurred in January 2025. An initial search strategy was outlined by two senior authors (A.J.L and M.J.K). The search was conducted according to the requirements of each database using the following search terms as well as their corresponding MeSH (Medical Subject Headings Terms): (near infrared autofluorescence OR NIRAF) AND (parathyroid surgery OR parathyroidectomy). The search was performed on the 25^th^ of July 2025.

### Data extraction

Duplicates of the records identified from the search were removed and the full titles of the studies were reviewed by two independent authors. The full texts of the studies were then assessed against the eligibility criteria. Any discrepancies in opinion were arbitrated by a third author (S.A.E). Basic clinicopathological parameters were collected from the studies. Study characteristics collected to meet the outcomes of this study included setting of the study, NIRAF device used, surgery type, surgeon experience, cure rates, operative time and number of PGs identified. The quality of the studies was assessed using the Cochrane Risk of Bias 2 tool for randomized control trials and the Newcastle-Ottawa Quality Assessment Scale for cohort studies (16, 17).

### Statistical analysis

Descriptive statistics were utilized as appropriate to delineate the basic clinicopathological parameters of the study. Mean operating time and mean number of parathyroid glands identified were depicted as Mean Differences (MD) with 95% confidence intervals (95% CI) as appropriate. Cure rates were depicted as risk ratios with 95% CI as appropriate. Results were deemed significant when p < 0.05 level, or the 95% CI did not incorporate a value of 1. Statistical analysis in this systematic review was conducted using R statistical software (18).

## Results

### Search

The search yielded 1,139 articles from the aforementioned databases. Following the removal of 334 duplicates, the titles of 805 records were screened based on their titles and abstract. Thereafter, 79 studies were subsequently reviewed in their full-text against the eligibility criteria. Of these, 3 studies met the inclusion criteria and were included in the systematic review and meta-analysis (12, 19, 20). A PRISMA flow-chart delineating the search process is shown in **Figure 1**.

**Figure 1:**
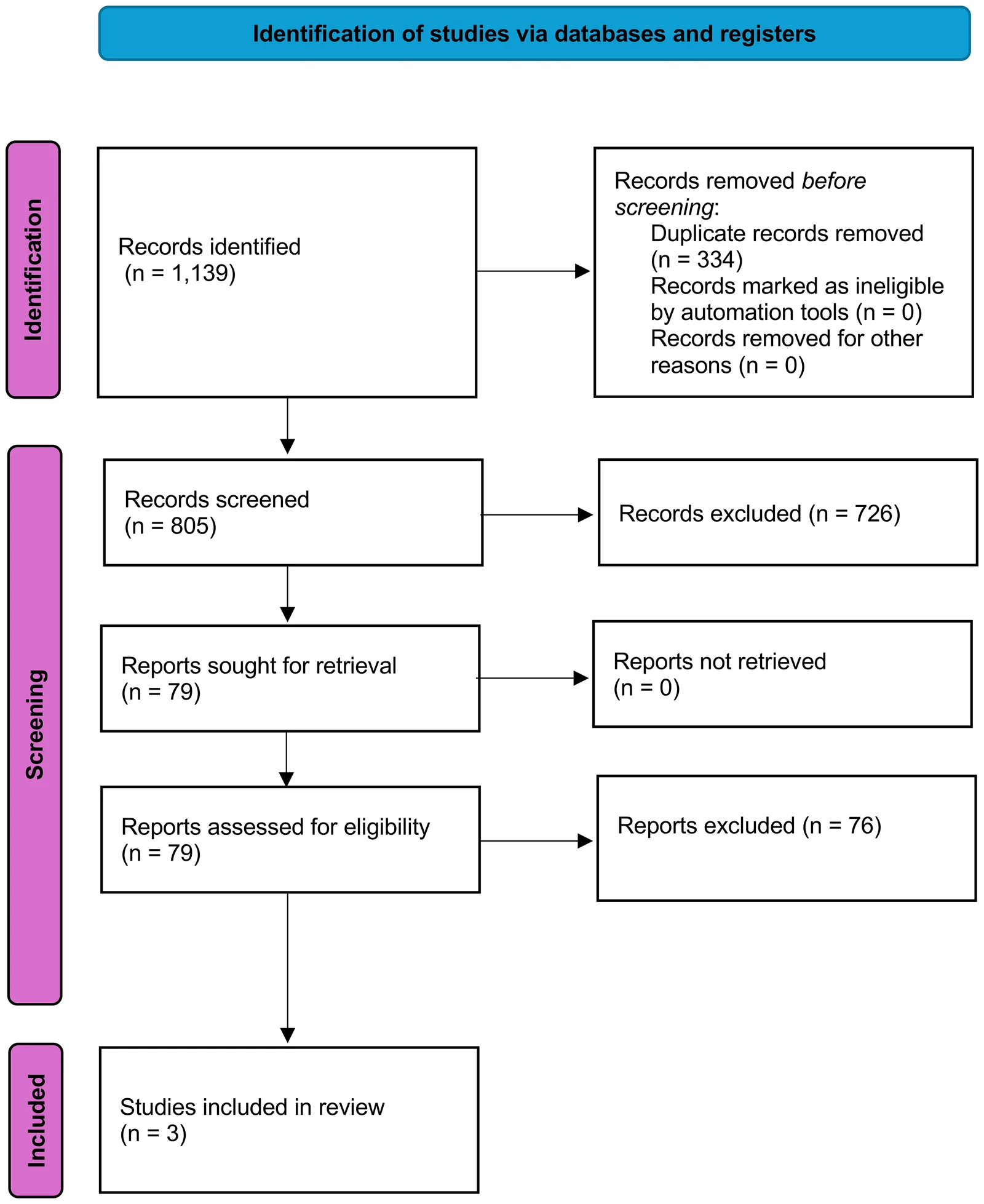
PRISMA flow diagram for systematic review.

### Study characteristics and clinicopathological data

Two of the three included studies were RCTs and one study was an observational cohort study. The studies were published between 2023 and 2025. All three studies included outcomes on cure rates, operative time and number of parathyroid glands identified. Data on a total of 522 patients was available. The number of participants in the studies ranged from 160 to 230 patients. Devices used included NIRAF probe (PT eye, Medtronic) and NIRAF imaging (Fluobeam 800, Elevision IR, Fluobeam LX). There were 196 (37.5%) patients in the NIRAF arm and 326 (62.5%) patients in the control arm. All studies had at least a 6 month follow-up duration. Study characteristics are outlined in **Table 1**.

**Table 1:** Characteristics of included studies.

Study	Journal	Setting	Design	Device	Population	Surgery type	Surgeon experience	Localized pre-op	Sample size	NIRAF	Control	Follow up (months)	Primary outcome
Kiernan et al., 2023 (12)	Annals of Surgery	Vanderbilt University Medical Center, USA	RCT	NIRAF probe (PT eye, Medtronic)	PHPT	Focused or BNE based on localization	Senior > 20 years and junior < 5 years	89	160	80	80	6	Persistent/failure PHPT, PGs identified, operative time, FS sent, permanent hypoparathyroidism, cure rates
Frey et al., 2025 (19)	Surgery	CHU Nantes, France	RCT	NIRAF imaging (Fluobeam 800 Getinge)	Mild PHPT	Planned BNE	> 15 years	126	124	59	64	6	Operating time, PGs identified/resected, complication rates, cure rates
Pannu et al., 2023 (20)	Frontiers in Endocrinology	Sheffield, UK	Cohort	NIRAF imaging (Elevision IR, subset Fluobeam LX)	PHPT	Focused or BNE based on localization	Consultant endocrine surgeon	156 localized on US 123 localized on Sestamibi	230	50	180	6	Cure rates, operating times, fluorescence patterns, glands identified

NIRAF, near infrared autofluorescence; PHPT, primary hyperparathyroidism; BNE, bilateral neck exploration; PG, parathyroid gland; UK, United Kingdom; USA, United States of America; CHU, Centre Hospitalier Universitaire; RCT, randomized controlled trial; US, ultrasound; FS, frozen section.

Of the overall cohort, 422 of the patients were female (78.9%, 422/522). Overall mean age of the included patients was 60.4 ± 10.8 years. Pooled pre-operative calcium could not be calculated as two studies used adjusted calcium for albumin and one study used total calcium. Mean pre-operative PTH for the three studies was 125.2 ± 80.3 pg/ml. Basic patient demographics are outlined in **Table 2**.

**Table 2:** Patient demographics.

Study	N	NIRAF	Control	Female Sex	Mean total calcium mmol/L (SD)	Mean adjusted calcium mmol/L (SD)	Mean age in years (SD)	Mean preop PTH in pg/ml (SD)
Kiernan et al.	160	80	80	125	2.77 (0.18)	N/R	58 (14.5)	157.9 (112.7)
Frey et al.	132	66	66	113	N/R	2.63 (0.11)	64 (12)	86.1 (11.5)
Pannu et al.	230	50	180	174	N/R	2.41 (0.18)	60.5 (5.8)	125 (75.6)
Total/Mean (SD)	522	196	326	412			60.4 (10.8)	125.2 (80.3)

NIRAF, near infrared autofluorescence; SD, standard deviation; PTH, parathyroid hormone; N/R, not reported.

### Quality assessment

Risk of bias assessment was performed using the Cochrane Risk of Bias 2 (RoB 2) tool for the two randomized controlled trials and the Risk of Bias in Newcastle-Ottawa Quality Assessment Scale for the observational cohort study (16, 17). The two RCTs were assessed as having a low risk of bias (**Figure 2**). The observational cohort study was assessed as high quality.

**Figure 2:**
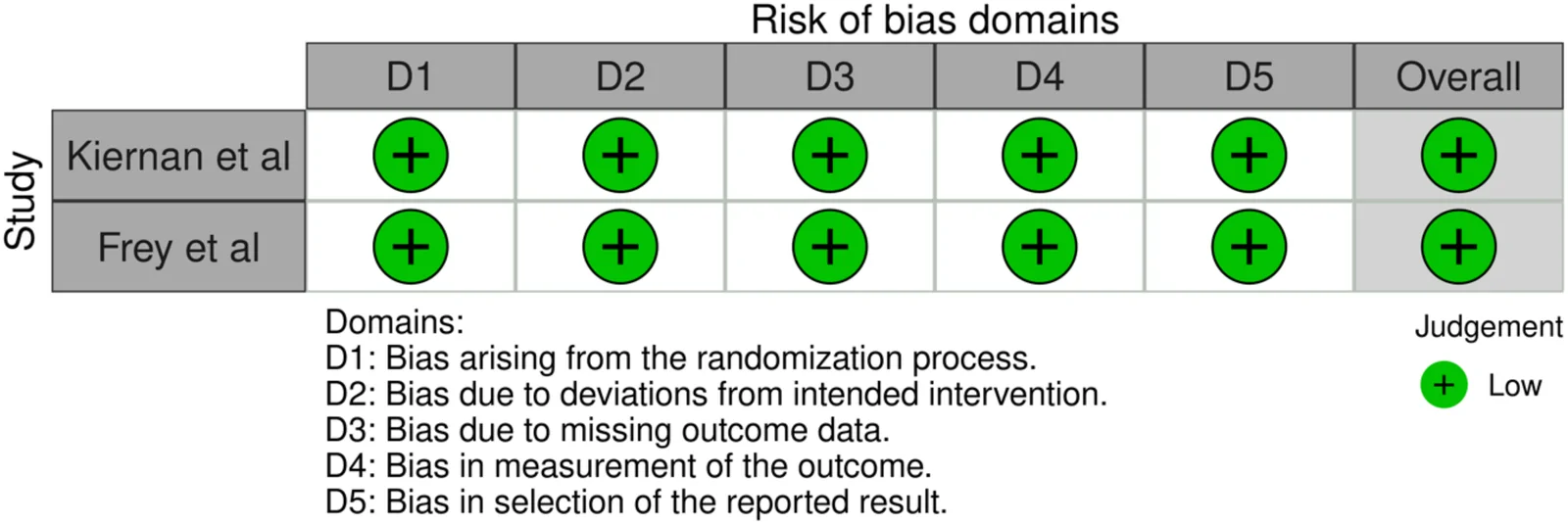
Cochrane risk of bias 2 graphical representation for RCTs.

### Primary outcome – cure rates

All three studies reported on cure rates following parathyroidectomy (**Figure 3**). Frey et al. and Pannu et al. defined cure as normalization of serum calcium and PTH at 6 months whereas Kiernan et al. did not explicitly mention cure rates but included rates of persistent hyperparathyroidism at last follow up (12, 19, 20). The meta-analysis using a random effects model demonstrated a non-significant increase in cure rates in in the NIRAF group compared to the control (RR 1.06, 95% CI: 0.97-1.15, z = 1.25, p = 0.21), though the confidence interval in the random effects model suggest uncertainty in the overall effect. The fixed effects models showed an increase in cure rates (RR = 1.06, 95% CI 1.02-1.10, z = 3.03, p = 0.002) which was statistically significant. However, due to the substantial heterogeneity across these studies, the random effects model is preferred for interpretation. At the individual study level, Kiernan et al. reported near identical cure rates between the groups (RR 1.01, 95% CI 0.99-1.04). Frey et al. observed a significant improvement in the NIRAF group (RR 1.18, 95% CI 1.06-1.31). Pannu et al. showed a marginal and non-significant benefit (RR 1.02, 95% CI 0.97-1.07).

**Figure 3:**
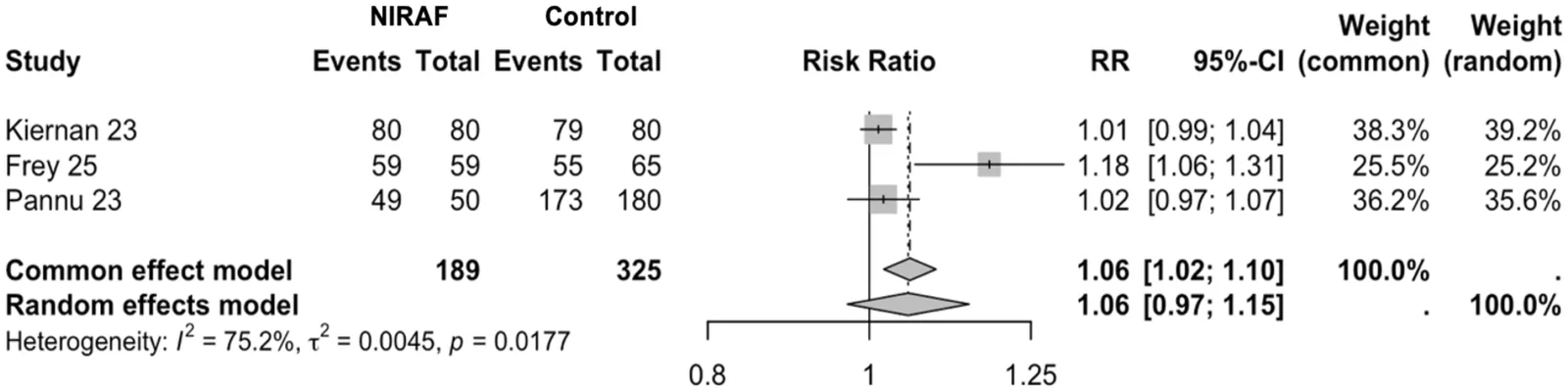
Forrest plot of cure rates. SD, standard deviation; R, risk ratio; CI, confidence interval.

There was a high degree of heterogeneity across the studies (I^2^ = 75.2%, p = 0.0117). This may be accounted for by variation in patient populations, surgical technique, surgeon experience, device used or follow up definitions.

### Secondary outcomes

#### Operating time

Mean operating time was reported across all three of the included studies (**Figure 4**). The random effects model meta-analysis showed a mean reduction in operative time of 2.12 minutes in the NIRAF group compared to the control (MD -2.12 minutes, 95% CI -4.76-9.0, z = 0.74, p = 0.55). However, both the 95% confidence interval and the p-value did not reach statistical significance. None of the studies showed a statistically significant difference in mean operative time at the individual study level.

**Figure 4:**
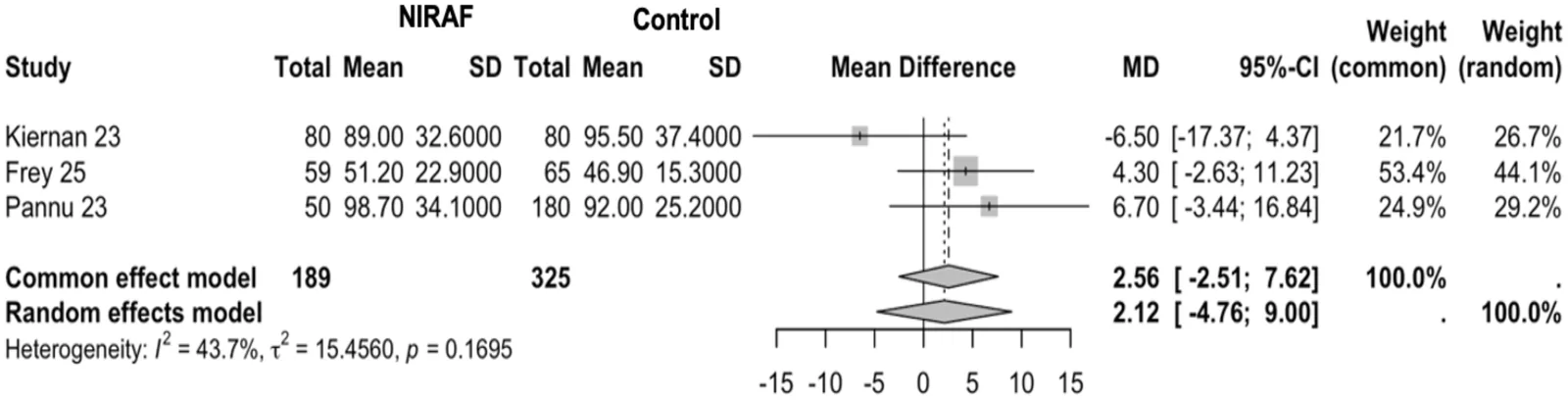
Forrest plot of mean operative times. SD, standard deviation; RR, risk ratio; CI, confidence interval.

The degree of heterogeneity was moderate (I^2^ = 43.7%) but the p-value was non-significant (p = 0.1695), suggesting some variation but an acceptable level of consistency across the three studies.

### Number of parathyroid glands identified

All of the three included studies reported on the mean number of parathyroid glands identified intra-operatively per patient (**Figure 5**). The random effects meta-analysis demonstrated a non-significant mean increase of 0.12 parathyroid glands identified per patient in the NIRAF compared to the controls (MD 0.12, 95% CI -0.21-0.46, z= 0.74, p = 0.46). At the individual study level, only Kiernan et al. showed a statistically significant increase in parathyroid glands identified (MD 0.40, 95% CI 0.12-0.68). There was moderate heterogeneity across the studies (I2 = 63.2%, p = 0.0681) which may be due to differences in surgeon experience and types of surgery across the studies.

**Figure 5:**
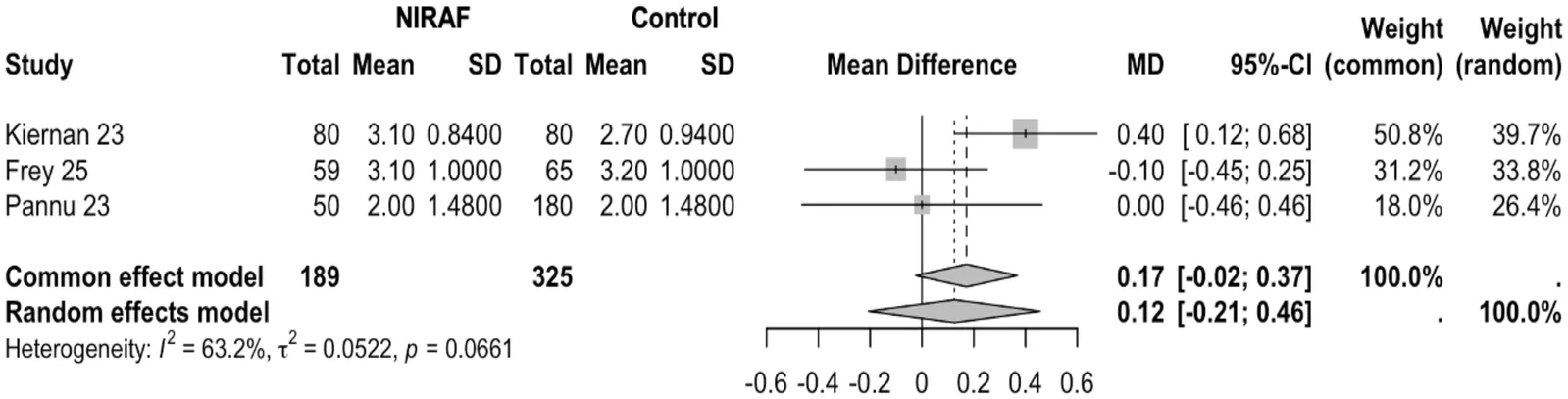
Forrest plot of mean number of parathyroid glands identified. SD, standard deviation; RR, risk ratio; CI, confidence interval.

## Discussion

This systematic review and meta-analysis presented the first synthesis to date of current comparative data regarding the use of NIRAF and visual inspection in parathyroid surgery for PHPT. Our findings suggest a small improvement in cure rates and a small improvement in intraoperative parathyroid gland identification with NIRAF, although these did not reach statistical significance with random-effects models. The differences in operative time with the use of NIRAF were negligible and non-significant.

Definitive cure of PHPT is heavily dependent on successful localization and excision of the hyperfunctioning parathyroid tissue. The use of NIRAF as a novel tool to support parathyroid gland identification by utilizing the intrinsic autofluorescent properties of the gland is promising without the requirement for radiation or contrast agents and allows real-time visualization (10). Several devices are now commercially available offering probe-based or camera-based guidance. In the setting of total thyroidectomy, NIRAF may reduce the risk of post-operative hypocalcaemia and post-operative hypoparathyroidism by aiding in preservation of parathyroid glands intra-operatively (21).

In this meta-analysis, NIRAF use was associated with a modest increase in cure rates with a pooled RR of 1.06 (95% CI 0.97-1.15, p = 0.21). While this trend favored NIRAF, it did not reach statistical significance on the random effects model and there was significant heterogeneity (I^2^ = 75.2%) present. Frey et al. reported a significant benefit (RR 1.18, 95% CI 1.06–1.31), whereas Kiernan et al. and Pannu et al. reported more modest effects. The difference in the effect of NIRAF on the cure rate across the studies may be due to variation in surgical technique, experience or technology used (probe versus imaging). Not all studies had clear, uniform definitions of persistent disease or cure, which may contribute to clinical heterogeneity. Overall, these findings suggest that the use of NIRAF may be associated with a small improvement in cure rates although this did not reach statistical significance in the random effects model. The heterogeneity observed also warrants caution in the interpretation of this result.

NIRAF was associated with a small, non-significant increase in the number of parathyroid glands identified (MD 0.12, 95% CI -0.21-0.46, p = 0.46). Only Kiernan et al. achieved statistical significance (MD 0.40, 95% CI 0.12-0.68). The subjectivity of gland identification makes this outcome vulnerable to bias.

Our study demonstrated no significant difference in mean operating time (MD 2.12 minutes, 95% CI -4.76-9.00, p = 0.46). This may be due to practical aspects of interpreting fluorescence and switching views offsetting any potential gains. None of the studies reported any significant learning curve effect with the use of NIRAF technology, but it is plausible in the early adoption phase that user unfamiliarity may impact any potential reduction in operative time. Although the pooled result favored a marginal decrease in operative time for NIRAF, this was not statistically significant. This indicates that the impact of NIRAF on operative efficiency is likely minimal, similar to the conclusions of the individual studies included (12, 19, 20).

In terms of methodology, the inclusion of two RCTs and one high quality observational cohort study allows for relative robustness. Quality appraisal via RoB 2 and NOS showed low risk of bias overall. However, the small number of studies and the heterogeneity in the outcomes limit broad generalizability. The outcomes of cure rates and mean operative time are objective, especially when using a biochemical definition of cure. Whereas, parathyroid identification is more subjective and prone to detection bias.

Additional limitations include the variation in devices used in the studies (probe based vs image based NIRAF) and lack of data on long-term recurrence or cost-effectiveness. The economic burden of NIRAF in the setting of total thyroidectomy has been recently published which noted an additional cost of approximately $639 per surgery and may be economically feasible for the prevention of post operative hypoparathyroidism in thyroid surgery (22).

Despite these caveats, the potential of NIRAF to assist in intraoperative decision making in parathyroid surgery is promising. Future research should involve large multicenter RCTs with standardized outcome definitions and device modalities. Multi-modal approaches combining NIRAF with other intra-operative modalities such as reflectance spectroscopy may enhance utility and warrant further investigation (23).

## Conclusion

Near-infrared autofluorescence is a technically feasible and safe adjunct to support parathyroid gland identification during parathyroidectomy for PHPT. While our systematic review and meta-analysis suggests possible small benefits in cure rates and gland identification, these effects did not achieve statistical significance in the random effects model. The use of NIRAF did not appear to offer a meaningful decrease in operative time. Further high quality, multicentered RCTs are essential before NIRAF can be utilized as a tool that improves surgical outcomes in the setting of parathyroidectomy for PHPT when compared with traditional techniques.

## Data Availability

The raw data supporting the conclusions of this article will be made available by the authors, without undue reservation.

## References

[1] Pokhrel B Leslie SW Levine SN . Primary hyperparathyroidism. Treasure Island (FL: StatPearls Publishing (2024).

[2] Press DM Siperstein AE Berber E Shin JJ Metzger R Monteiro R . The prevalence of undiagnosed and unrecognized primary hyperparathyroidism: a population-based analysis from the electronic medical record. Surgery. (2013) 154:1232–7. doi: 10.1016/j.surg.2013.06.051 24383100

[3] Baj J Sitarz R Łokaj M Forma A Czeczelewski M Maani A . Preoperative and intraoperative methods of parathyroid gland localization and the diagnosis of parathyroid adenomas. Molecules. (2020) 25. doi: 10.3390/molecules25071724 32283730 PMC7181220

[4] Kose E Rudin AV Kahramangil B Moore E Aydin H Donmez M . Autofluorescence imaging of parathyroid glands: an assessment of potential indications. Surgery. (2020) 167:173–9. doi: 10.1016/j.surg.2019.04.072 31526579

[5] Salman R Sebaaly MG Wehbe MR Sfeir P Khalife M Al-Kutoubi A . Preoperative localization of mediastinal parathyroid adenoma with intra-arterial methylene blue. Cardiovasc Interventional Radiol. (2017) 40:953–6. doi: 10.1007/s00270-017-1578-4 28138727

[6] Spartalis E Ntokos G Georgiou K Zografos G Tsourouflis G Dimitroulis D . Intraoperative indocyanine green (ICG) angiography for the identification of the parathyroid glands: current evidence and future perspectives. In Vivo. (2020) 34:23–32. doi: 10.21873/invivo.11741 31882459 PMC6984100

[7] Rubello D Pelizzo MR Boni G Schiavo R Vaggelli L Villa G . Radioguided surgery of primary hyperparathyroidism using the low-dose 99mTc-sestamibi protocol: multiinstitutional experience from the Italian Study Group on Radioguided Surgery and Immunoscintigraphy (GISCRIS). J Nucl Med. (2005) 46:220–6. doi: 10.1007/978-0-387-48894-3_28 15695779

[8] Barczyński M Gołkowski F Nawrot I . The current status of intraoperative iPTH assay in surgery for primary hyperparathyroidism. Gland Surg. (2015) 4:36–43. doi: 10.3978/j.issn.2227-684X.2015.01.01 PMC4321047 25713778

[9] Di Marco AN Palazzo FF . Near-infrared autofluorescence in thyroid and parathyroid surgery. Gland Surg. (2020) 9:S136–46. doi: 10.21037/gs.2020.01.04 32175254 PMC7044090

[10] McWade MA Paras C White LM Phay JE Solórzano CC Broome JT . Label-free intraoperative parathyroid localization with near-infrared autofluorescence imaging. J Clin Endocrinol Metab. (2014) 99:4574–80. doi: 10.1210/jc.2014-2503 25148235 PMC4255111

[11] Kim DH Lee S Jung J Kim S Kim SW Hwang SH . Near-infrared autofluorescence-based parathyroid glands identification in the thyroidectomy or parathyroidectomy: a systematic review and meta-analysis. Langenbecks Arch Surg. (2022) 407:491–9. doi: 10.1007/s00423-021-02269-8 34322746

[12] Kiernan CM Thomas G Patel A Fan R Ye F Willmon PA . Does the use of probe-based near-infrared autofluorescence parathyroid detection benefit parathyroidectomy?: a randomized single-center clinical trial. Ann Surg. (2023) 278:549–58. doi: 10.1097/sla.0000000000005985 37389537 PMC10836830

[13] Rossi L De Palma A Papini P Chicas Vasquez M Cetani F Ambrosini CE . Near-infrared autofluorescence pattern in parathyroid gland adenoma. Surg Endosc. (2024) 38:6930–7. doi: 10.1007/s00464-024-11314-8 39382656 PMC11525238

[14] Page MJ McKenzie JE Bossuyt PM Boutron I Hoffmann TC Mulrow CD . The PRISMA 2020 statement: an updated guideline for reporting systematic reviews. Bmj. (2021) 372:n71. doi: 10.31222/osf.io/v7gm2 33782057 PMC8005924

[15] Richardson WS Wilson MC Nishikawa J Hayward RS . The well-built clinical question: a key to evidence-based decisions. ACP J Club. (1995) 123:A12–3. doi: 10.7326/ACPJC-1995-123-3-A12 7582737

[16] Higgins JP Altman DG Gøtzsche PC Jüni P Moher D Oxman AD . The Cochrane Collaboration's tool for assessing risk of bias in randomised trials. Bmj. (2011) 343:d5928. doi: 10.1136/bmj.d5928 22008217 PMC3196245

[17] Wells G O'Connell D . The newcastle–ottawa scale (NOS) for assessing the quality of nonrandomised studies in meta‐ Analyses.

[18] Team RC . R: A Language and Environment for Statistical Computing. Vienna, Austria: R Foundation for Statistical Computing (2024).

[19] Frey S Bannani S Caillard C Le Bras M Drui D Ansquer C . Parathyroid near-infrared autofluorescence use for parathyroidectomy in mild primary hyperparathyroidism: results from a randomized monocentric trial. Surgery. (2025) 177:108878. doi: 10.1016/j.surg.2024.05.062 39443204

[20] Pannu AY O'Connor-Manson MR Wyld L Balasubramanian SP . Near-infrared fluorescent imaging for parathyroid identification and/or preservation in surgery for primary hyperparathyroidism. Front Endocrinol (Lausanne). (2023) 14:1240024. doi: 10.3389/fendo.2023.1240024 38111703 PMC10726132

[21] Javed A Alburaiki A Sharma N De M Garas G Ahmad I . Utilisation of near infrared autofluorescence in parathyroid identification during thyroidectomy: a systematic review and meta-analysis of randomised controlled trials. Clin Otolaryngol. (2025) 50:609–18. doi: 10.1111/coa.14313 40186524

[22] Serrao-Brown HG Papachristos AJ Sidhu SB . Cost-benefit analysis of intraoperative autofluorescence for parathyroid identification. JAMA Otolaryngology–Head Neck Surg. (2025) 151:313–8. doi: 10.1001/jamaoto.2024.5185 39946100 PMC11826435

[23] Tseregorodtseva P Budylin G Korneva N Kim I Shiriaev S Yakimov B . Parathyroid glands viability assessment and identification during surgery with multimodal diffuse reflectance spectroscopy and NIR autofluorescence probe. Sci Rep. (2025) 15:22097. doi: 10.1038/s41598-025-04059-4 40592993 PMC12216779

